# AVB81: A Fine-Grained Audiovisual Dataset and Benchmark for Bird Classification in the Wild

**DOI:** 10.3390/ani16142130

**Published:** 2026-07-09

**Authors:** Xiaodong Zhao, Shanshan Xie, Jiangjian Xie

**Affiliations:** 1 Henan Forestry Information Engineering Technology Research Center, Henan Forestry Vocational College, Luoyang 471002, China; davidzhao30@163.com; 2 Multimodal Eco Data Intelligence Analysis Laboratory, School of Technology, Beijing Forestry University, Beijing 100083, China; xieshanshan@bjfu.edu.cn; 3 State Key Laboratory of Efficient Production of Forest Resources, Beijing Forestry University, Beijing 100083, China

**Keywords:** audiovisual fusion, fine-grained bird classification, wildlife intelligent monitoring

## Abstract

Automated bird monitoring plays a crucial role in ecoinformatics and biodiversity conservation. Despite significant advancements in fine-grained visual classification, targets in complex wild environments frequently encounter interferences such as long capture distances, severe visual occlusion, and strong background noise, rendering single-modal perception highly susceptible to performance bottlenecks. To advance research in audiovisual multimodal bird classification, this paper introduces AVB81, a multimodal dataset tailored for fine-grained bird recognition. Covering 81 bird species in North America, the dataset comprises 3247 fixed-length 10 s field videos, meticulously supplemented with 5418 independent audios and 7083 high-quality static images. Based on this dataset, we systematically conduct single-modal performance evaluations and cross-paradigm audiovisual fusion experiments, establishing a comprehensive and in-depth multimodal evaluation benchmark. The experimental results demonstrate that in video classification tasks, audiovisual multimodal fusion methods significantly outperform single-modal baselines. Notably, the mid-fusion strategy based on deep semantic spaces achieves the optimal performance. Furthermore, the experiments objectively reveal the formidable challenges associated with audio recognition within videos captured in complex wild habitats. Overall, AVB81 exhibits exceptional adaptability for multimodal research, providing a highly challenging evaluation benchmark for multimodal semantic modeling in complex scenarios and laying a solid data foundation for the development and validation of next-generation intelligent ecological monitoring systems.

## Simple Summary

Wild bird monitoring is frequently hindered by factors such as long distances, visual occlusions, and background noise interference, rendering traditional classification methods that rely solely on visual or acoustic cues inherently limited. To advance bird classification research that integrates visual and acoustic information, we construct a comprehensive dataset, AVB81, which encompasses videos, audios, and images across 81 bird species. Experimental results not only demonstrate the exceptional applicability of the AVB81 dataset in multimodal research, but also reveal that synergizing visual and audio information within videos significantly enhances recognition accuracy compared to utilizing unimodal visual or audio methods alone. Furthermore, the findings objectively highlight the formidable challenges associated with audio recognition in wild monitoring videos. We believe this dataset serves as an excellent benchmark for testing and refining sophisticated automated bird recognition technologies. This work establishes a solid data foundation for the development of more intelligent avian monitoring systems and is pivotal for deepening our understanding of ecosystems and conserving global biodiversity.

## 1. Introduction

Fine-grained visual classification, which aims to distinguish sub-categories with high similarity (such as different bird species) [1], has long been a pivotal research direction in computer vision. In recent years, deep learning methods have significantly enhanced image classification performance, leading to substantial progress in fine-grained visual bird recognition [2]. However, in real-world ecological monitoring applications, birds are typically small-scale targets frequently obscured by tree canopies and subjected to complex illumination. Consequently, relying solely on the visual modality often encounters technical bottlenecks. In this context, acoustic signals from bird vocalizations, which are unconstrained by line-of-sight and lighting limitations, serve as a critical complement to break this impasse. The introduction of audio not only aligns with the multi-dimensional observation patterns in biology, but also drives audiovisual multimodal fine-grained bird classification to become a core research hotspot for overcoming single-modality limitations and improving species monitoring accuracy [3,4].

Although multimodal fusion demonstrates immense potential, realizing this objective relies heavily on the support of high-quality data. Reviewing existing research, single-modal learning has benefited from the development of numerous mature datasets (such as CUB-200-2011 [5] and NABirds [6] in the image domain, as well as Birdset [7] and DB3V [8] in the audio domain), which have laid a solid foundation. Nevertheless, during the transition towards multimodal approaches, the limitations of data resources have gradually been exposed. Several existing bird video datasets (such as VB100 [9], YouTube-Birds [10], IBC127 [11], and FGB-AV [12]) lack unified category coverage across audio, images, and videos, making it difficult to support cross-modal semantic alignment research. SSW60 [13] is currently one of the more standardized and widely used multimodal bird classification datasets, having preliminarily verified the significance of multimodal fusion in enhancing bird classification performance.

However, real-world wild ecological monitoring environments are often characterized by high uncertainty. SSW60 still exhibits certain limitations in simulating complex realistic scenarios and handling multimodal consistency. First, regarding species selection, the dataset presents a specific habitat bias: it primarily focuses on birds with a high probability of appearing around “fixed bird feeders” in the Ithaca area of New York. This confines the category distribution to common birds adapted to human-adjacent environments, thereby reflecting a relatively restricted ecological diversity. Second, its strategy of forcibly filtering actively vocalizing clips via an audio recognition model (Merlin Sound ID), while boosting experimental performance, largely excludes audiovisual asynchronous or silent observation samples that are extremely prevalent in wild environments, thereby diminishing the inherent randomness of the wild. Furthermore, in terms of visual presentation, this dataset fails to adequately encompass extreme visual challenges—such as long-distance observations and small target scales—which are exceedingly common in actual field wildlife monitoring.

To this end, this paper constructs AVB81, a novel fine-grained bird benchmark dataset characterized by multimodal completeness, ecological expansiveness, and realistic visual challenges, aiming to provide a vital supplement to existing audiovisual multimodal research. Covering 81 bird species, the dataset achieves the systematic integration of multi-source heterogeneous data—including video, audio, and images—under a unified category system. Specifically, in the video modality, we extract raw video samples spanning 81 species from the VB100 dataset and standardize them into 10 s clips. This process yields 3247 samples that fully preserve high-difficulty spatial-temporal dynamic information under conditions such as long-distance observation, drastic target scale variations, camera motion, and complex background interference. In the audio modality, we extract 5418 independent 10 s recordings of the same species from the Xeno-canto database [14]. This captures high-quality and diverse inherent species acoustic features, providing robust acoustic feature support for audiovisual fusion models. In the image modality, we integrate 7083 corresponding static samples from Birdsnap [15], furnishing high-resolution static spatial texture support for the models. Through such meticulous multimodal data construction, AVB81 offers a highly challenging and flexible research foundation for fine-grained joint audiovisual classification and cross-modal learning. Importantly, unlike conventional multimodal benchmarks that rely on strict instance-level alignment, AVB81 adopts a hybrid construction paradigm. It therefore constitutes a weakly aligned ecological multimodal learning setting that better reflects real-world sensing conditions.

Based on this dataset, we further conduct a systematic evaluation of the fine-grained audiovisual multimodal classification task. We establish an evaluation benchmark encompassing various mainstream deep learning architectures and comprehensively investigate the effectiveness of different cross-modal fusion strategies. Experimental results indicate that the classification performance of multimodal synergistic modeling significantly surpasses that of single-modality methods. This verifies the efficacy of audiovisual information complementarity and provides new benchmarks and directions for future intelligent wildlife monitoring and multimodal data fusion research.

The main contributions of this paper are summarized as follows:

(1) Construction of the AVB81 multimodal benchmark by systematically integrating video, image, and audio data from existing public repositories under a unified category system. This provides a rigorously validated data foundation for studying multimodal learning in authentic wild scenarios.
(2) Establishment of an audiovisual fusion evaluation benchmark and systematic analysis. We uniformly implement and evaluate multiple deep learning models on this dataset, systematically comparing typical strategies such as early fusion, mid-fusion, and late fusion. This establishes a comprehensive reference for multimodal benchmarks.

## 2. Related Work

### 2.1. Bird Fine-Grained Visual and Audio Datasets

The computer vision community has constructed numerous fine-grained image datasets. Among them, bird datasets (such as iNat2021 [16] and Birds-525 [17]), characterized by real-world attributes such as long-tailed distributions, high inter-species visual similarity, and complex backgrounds, are extensively utilized to evaluate the performance of fine-grained classification algorithms. In the audio domain, datasets for environmental sound classification and species recognition have also witnessed substantial development. For instance, Xeno-canto [14], the world’s largest and most active wild bird sound-sharing community, along with the BirdCLEF2023 [18] series of large-scale birdsong databases, has provided crucial support for acoustic modeling.

However, these datasets are typically confined to a single modality. Visual and audio data generally exist in isolation, lacking cross-modal pairing relationships under a unified category system. Such modality fragmentation severely limits the models’ capability to capture deep semantic associations between visual and acoustic information, thereby constraining the further advancement of audiovisual multimodal fine-grained recognition technologies.

### 2.2. Bird Fine-Grained Video Datasets

Video serves as a vital source of animal ecological information [19], containing rich spatial-temporal dynamic features and possessing an inherent audiovisual co-occurrence attribute. Existing video datasets, represented by VB100 [9] and YouTube-Birds [10], are primarily contributed by professional observers or open-source communities. The advantage of such data lies in its high fidelity to real-world wild challenges, encompassing intense camera tracking motions, extremely small target scales, and complex habitat backgrounds. Furthermore, the WetlandBirds [19] dataset expands the dimensions of bird species recognition and behavioral information detection. However, when constructing benchmarks, the aforementioned datasets are frequently confined to purely visual classification tasks. Due to the lack of explicit task definitions for audiovisual synergistic modeling and high-quality single-modal annotation support, the audio information embedded in videos has long been marginalized into a state of “data stored but unmodeled”, failing to fully unleash the potential of audiovisual multimodal synergistic representation.

SSW60 [13] is a representative work in the field of audiovisual fusion. By providing aligned video clips, audio records, and static image data, it offers a standardized evaluation platform for multimodal fusion algorithms. Nonetheless, it sacrifices the true randomness of the natural environment to some extent, and its species distribution is limited to a specific, relatively small geographic area. AVB81 aims to bridge the gap between the two aforementioned types of datasets. It not only inherits the highly challenging wild characteristics from VB100, but also introduces high-quality independent recordings from Xeno-canto and static images from Birdsnap under a unified category system. Unlike SSW60, AVB81 does not enforce mandatory audiovisual matching filtering during its construction. It is designed to support the modeling of modality inconsistency, signal asynchrony, and scene complexity in realistic monitoring, thereby propelling fine-grained video classification towards more authentic and complex ecological monitoring scenarios. Table 1 provides a comprehensive comparison between AVB81 and existing bird-related datasets.

### 2.3. Audiovisual Multimodal Fusion

Audiovisual multimodal fusion is dedicated to developing models capable of jointly understanding and exploiting visual and audio information, aiming to leverage the complementarity between visual and audio data to enhance the performance of downstream tasks [20]. In the realm of fine-grained bird species recognition, multimodal fusion methods that combine acoustic and visual features have emerged as a key approach to resolving recognition challenges in complex wild scenarios.

With the evolution of deep learning technologies, researchers have explored diverse audiovisual fusion mechanisms across different network stages. Early studies predominantly focused on early or late fusion strategies. The former directly fuses data from respective modalities before feeding it into models for feature extraction and decision-making [21], whereas the latter combines the discriminative results from each modality using specific fusion rules to generate the final output decision [22]. In recent years, to better balance modality-specific representations and cross-modal interactions, mid-fusion has gradually emerged as the mainstream approach. Such methods typically feed visual and audio modalities into independent backbone networks for encoding. Subsequently, in a shared feature space, they construct a joint audiovisual representation through operations such as feature concatenation and element-wise addition [23], thereby achieving the joint optimization of cross-modal information.

In summary, early, middle, and late fusion strategies have distinct emphases regarding feature interaction mechanisms. To systematically verify the capability of our constructed multimodal dataset, AVB81, in supporting multimodal modeling for fine-grained bird recognition tasks, we comprehensively implement and incorporate the aforementioned representative audiovisual fusion methods during the experimental phase. By conducting systematic baseline experiments across various fusion levels and strategies on the AVB81 dataset, we not only thoroughly validate the efficacy of AVB81 in supporting diverse fusion strategies, but also conduct an in-depth analysis of the performance disparities among these strategies in complex wild scenarios. This provides a solid data foundation and a comprehensive experimental reference for subsequent multimodal ecological monitoring research.

## 3. AVB81 Dataset

To support research in fine-grained audiovisual classification and cross-modal learning, this paper constructs the AVB81 dataset. This dataset achieves unified alignment among visual, audio, and video modalities at the species category level. Through multi-source data integration and standardized processing, it provides a systematic data foundation for multimodal fusion and alignment modeling.

### 3.1. Video Modality

Video data is sourced from the VB100 dataset, which covers 100 different North American bird species and contains 1416 videos with a median duration of approximately 32 s, documenting bird activity scenes in authentic wild environments. Captured by professional birdwatchers in the field, this footage exhibits visual characteristics such as long-distance observation, small target scales, significant posture and motion variations, and complex background interference. Although these interference factors from real-world environments exacerbate the difficulty of fine-grained classification, they also make the data more congruent with the requirements of practical automated ecological monitoring. Given that this paper aims to construct a dataset for deep audiovisual multimodal fusion, it is imperative to ensure the absolute completeness of data across all modalities at the category level. Because acquiring static images and high-quality independent audio for 19 bird species in the original dataset proved highly difficult, failing to meet the rigorous cross-modal pairing requirements, this study ultimately filters and retains 81 species with complete multimodal data dimensions. For the video data of these 81 bird species, to establish a unified spatial-temporal analysis benchmark, we segment the raw videos into 10 s clips with a stride of 10 s and discard clips shorter than 10 s. This standardizes the time window and mitigates interference caused by temporal length discrepancies. Such standardized processing facilitates enhanced comparability across samples and provides a consistent input structure for subsequent multimodal fusion modeling.

### 3.2. Audio Modality

To construct an independent and high-quality acoustic evaluation benchmark, audio data is sourced from Xeno-canto, a large-scale online sound database comprising over 200,000 bird recordings spanning 10,000 species globally. During the data screening process, we first perform species alignment based on the 81 bird species included in AVB81 and select audio samples with a quality rating of A or B. Given that the duration of raw audio typically ranges from tens of seconds to several minutes and contains complex environmental noise, we further apply denoising and uniformly crop the recordings into 10 s clips to ensure temporal consistency with the video modality. It is worth noting that this portion of the audio constitutes unpaired data; that is, its source is independent of the video modality. By stripping away the complex wild environmental noise present in videos, these recordings provide acoustic encoders with rich inherent acoustic priors of the species, thereby supporting research on cross-modal alignment and robustness.

### 3.3. Image Modality

Image data is derived from the Birdsnap dataset, finely annotated by professionals. This dataset contains 49,829 images covering 500 of the most common bird species in North America, with a relatively balanced inter-class data distribution (ranging from 69 to 100 images per category). Images in this dataset generally feature clear main targets and high resolution, providing stable appearance features for fine-grained visual modeling. In this study, we conduct rigorous reorganization targeting the 81 species included in the AVB81 benchmark, constructing a high-quality static image subset. Compared to the video modality, image data lacks temporal dimension information; however, its high-quality static representations serve as crucial visual priors, assisting the model in learning more discriminative feature representations. Within the multimodal learning framework, this modality helps compensate for visual information instability in videos caused by factors such as motion blur and scale variations, thereby enhancing the overall recognition performance.

**Figure 1:**
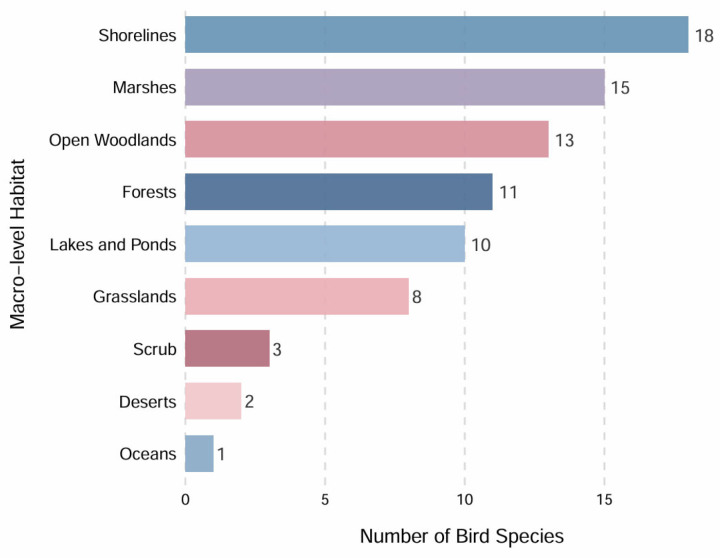
Macro-level habitat distribution of the 81 bird species in the AVB81 dataset.

In summary, the AVB81 dataset encompasses 81 bird species, comprising a total of 3247 videos, 5418 audios, and 7083 images. To comprehensively illustrate the ecological diversity of the dataset, the detailed geographic distribution and habitat information for all 81 species are provided in Appendix A (Table A1). Furthermore, Figure 1 presents the macro-level habitat distribution of the included species. The dataset covers a wide spectrum of environments—ranging from shorelines and marshes to forests and grasslands—which intrinsically introduces authentic real-world background variations (e.g., environmental noise and foliage occlusion) to the evaluation benchmark.

Regarding dataset splitting, tailored partitioning protocols are implemented across different modalities to strictly prevent potential data leakage and ensure evaluation validity. For the audio modality, a naive random split of the processed 10 s segments would introduce severe data leakage, as segments derived from the same continuous recording share identical ambient soundscapes and hardware fingerprints. To eliminate this risk, we enforced a strict recording-level isolation protocol. Specifically, after performing the initial 10 s temporal slicing, all segments were aggregated based on the unique recording IDs of their original continuous recordings from Xeno-canto. The 7:3 training and testing partition was then strictly executed at this macro recording level. This guarantees that all segments originating from the same continuous field recording are exclusively assigned to either the training or testing set, ensuring zero acoustic environmental overlap. Furthermore, for the video modality, we strictly adhered to the original video-level training and testing split protocols designated by the VB100 dataset. The 10 s temporal slicing was executed exclusively within these pre-isolated and disjoint video-level subsets. This natural hierarchical split ensures that all keyframes and audio tracks extracted from the same source video remain unified within the same set, thereby successfully avoiding any cross-contamination. Finally, for the image modality, images were randomly partitioned into training and testing sets with a 7:3 ratio, given the inherently discrete, mutually independent, and single-capture characteristics of Birdsnap static imagery. Through these rigorous source-disjoint strategies, AVB81 establishes a reliable, non-leaked benchmark that authentically assesses models’ generalization capabilities in complex wild environments.

**Figure 2:**
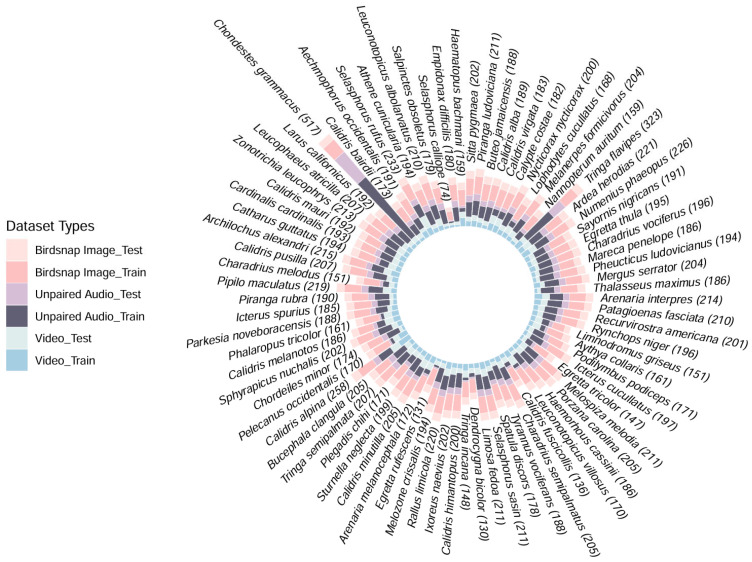
Distribution of sample sizes across different datasets for 81 bird species. Values in parentheses after each species name indicate the total number of samples combined across all six dataset split categories (Birdsnap Image, Unpaired Audio, and Video for both training and test sets).

To facilitate subsequent experimental descriptions and analyses, the independently collected audio modality is denoted as “Unpaired Audio”, the audio modality within the videos as “Video-Audio”, the visual modality within the videos as “Video-Frame”, and the static image modality as “Birdsnap Image”. The detailed species information is illustrated in Figure 2.

**Figure 3:**
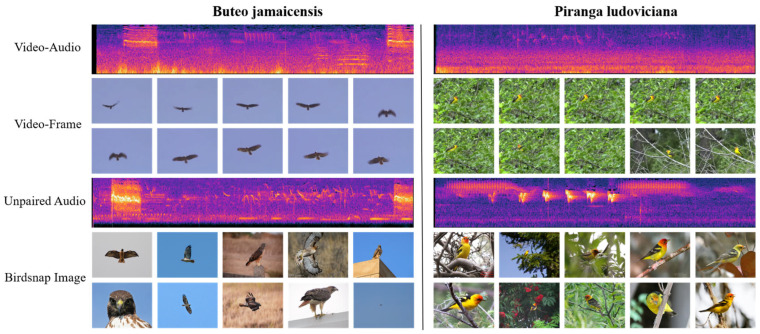
Examples of visual and audio modalities for detailed species information in the AVB81 dataset. For each species, the four rows display modal samples from: (1) Video-Audio (spectrogram derived from the audio track of a 10 s video); (2) Video-Frame (representative keyframe extracted from a 10 s video); (3) Unpaired Audio (spectrogram of a 10 s independent audio); and (4) Birdsnap Image. Note that multiple static Birdsnap images are presented to comprehensively demonstrate the rich fine-grained visual variations of the species across different capturing conditions, whereas a standard input sample during training consists of a single image.

Figure 3 presents sample data for species across different modalities in the AVB81 dataset. Through the unified curation and standardized processing of multi-source data, AVB81 ensures category consistency while simultaneously accommodating modality diversity and real-world complexity, thereby providing a challenging and practically valuable data foundation for fine-grained audiovisual classification and cross-modal fusion research.

### 3.4. Nature of Multimodal Alignment

The multi-modal relationships in AVB81 operate at two different levels: sample-level co-occurrence in videos and species-level alignment for independent data.

For the video modality (Video-Frame and Video-Audio), the visual and acoustic tracks encapsulate sample-level spatial-temporal co-occurrence, as they are captured from the exact same field recording sessions. However, in authentic wild environments, field videos are frequently plagued by signal asynchrony (e.g., a bird appears in the field of view but remains silent, or background noise completely masks the target vocalization). Because of the severe noises and misalignments inherent in these field videos, learning cross-modal representations directly and solely from video data is highly challenging. Therefore, the independent image and audio data (Birdsnap Image and Unpaired Audio) are introduced and aligned strictly at the species level (category level).

These independent resources are used to provide high-quality prior representations for the visual and audio encoders, thereby alleviating the learning difficulties caused by noise in wild video data. This design preserves realistic field videos for audiovisual learning while injecting cleaner modality-specific semantic priors for representation learning. Importantly, these independent resources are not involved in the audiovisual fusion or evaluation process. Instead, only synchronized Video-Frame and Video-Audio pairs extracted from the same videos are used for multimodal fusion and benchmarking, ensuring that all evaluated models operate on authentically paired audiovisual observations. This design establishes a weakly aligned ecological multimodal learning paradigm that deviates from conventional fully instance-aligned benchmarks by reflecting realistic cross-modal sensing conditions in wild environments.

## 4. Methodology

To systematically evaluate the multimodal modeling capability of AVB81 in the fine-grained bird recognition task, this study revolves around two core benchmarks: unimodal modeling analysis and audiovisual fusion.

### 4.1. Unimodal Modeling

In fine-grained audiovisual classification tasks, unimodal benchmarking serves as an essential prerequisite for validating dataset usability and analyzing the potential of multimodal fusion. Compared with directly constructing complex cross-modal interaction structures, unimodal modeling offers a more intuitive reflection of the data’s intrinsic feature quality, class separability, and the model’s representation capability across different modality information. Therefore, this paper first establishes a standardized unimodal evaluation framework encompassing mainstream deep learning architectures. By employing a unified end-to-end classification paradigm, we systematically analyze the learning performance of the AVB81 dataset under varying modalities, input modalities, and network configurations.

Concurrently, this phase of experiments establishes a unified and reproducible performance baseline for subsequent multimodal fusion experiments, thereby facilitating a more accurate analysis of the performance gains derived from various fusion strategies. Given that the primary objective of this study is to evaluate the adaptability of the AVB81 dataset under different structural hypotheses and feature modeling paradigms, we select widely representative deep models from current computer vision and audio processing domains as unimodal baselines. This yields a comprehensive evaluation system that covers both mainstream Convolutional Neural Network (CNN) and Transformer architectures.

#### 4.1.1. Visual Unimodal Modeling

For the visual modality, comparative experiments are conducted under two input settings—Birdsnap Image and Video-Frame—to evaluate the modeling difficulty of the dataset under varying visual information densities and temporal sampling conditions. Regarding model selection, this study encompasses both classic Convolutional Neural Network (CNN) and self-attention-based Transformer architectures.

First, concerning CNN architectures, ResNet-50 [24], as a classic deep residual network, exhibits exceptionally stable performance in local texture modeling and general feature extraction based on its convolutional structure. Meanwhile, to tackle the complex environmental interferences that frequently accompany fine-grained targets, the YOLOv11 [25] backbone network—leveraging its structural advantages in small target perception and multi-scale feature fusion—demonstrates stronger adaptability to scenarios involving long-distance capture and drastic target scale variations. Second, regarding Transformer architectures, ViT-B [26] discards traditional local convolutional operations and relies entirely on self-attention mechanisms to establish global long-range dependencies, thereby efficiently mining the global contextual information of images. Swin-T [27] introduces a hierarchical shifted window attention mechanism; while maintaining the model’s global perception capability, it effectively reduces computational complexity and significantly enhances the extraction ability for multi-scale targets and refined local features.

#### 4.1.2. Audio Unimodal Modeling

For the audio modality, modeling analyses are performed under two input settings—Unpaired Audio and Video-Audio—to systematically evaluate the audio representation capability of AVB81 across different acoustic scenarios and recording conditions. Regarding model selection, it similarly covers two mainstream acoustic modeling architectures: CNN-based and Transformer-based networks.

In terms of CNN architectures, the lightweight ResNet-18 [24] possesses a streamlined structure, enabling it to efficiently extract and fit local time-frequency patterns on spectrograms. CNN14 [28] serves as a universal benchmark model in the field of acoustic event classification; relying on its pre-training foundation over large-scale audio data, it can extract highly robust high-dimensional acoustic features, exhibit excellent classification performance when processing complex vocalizing targets. Regarding Transformer architectures, ViT-B is extended to process acoustic spectrograms, directly utilizing the self-attention mechanism to accurately capture the dependencies of audio signals across global time-frequency dimensions. Furthermore, HTSAT [29], as a hierarchical Transformer architecture specifically designed for audio spectrogram representation, can efficiently process complex overlapping soundscapes, demonstrating significant structural advantages in capturing multi-scale acoustic events and long-temporal signal features.

#### 4.1.3. Feature Representation and Classification

Despite the variations in network architectures, feature extraction paradigms, and modeling mechanisms among different visual and audio models, the overall pipeline consistently comprises two stages: feature extraction and classification prediction. Therefore, we provide a unified formulation for the feature representation and classification processes within the unimodal tasks. Let the visual and audio inputs be denoted as $x_{v}$ and $x_{a}$, respectively. Specifically, based on the experimental settings, the visual input $x_{v}$ can be instantiated as Video-Frame or Birdsnap Image, while the audio input $x_{a}$ corresponds to the Mel spectrogram representation of either Unpaired Audio or Video-Audio.

The visual and audio encoders are denoted as $\phi_{v}(\cdot )$ and $\phi_{a}(\cdot )$, respectively. Here,$\;\phi_{v}(\cdot )$ and $\phi_{a}(\cdot )$ represent the visual and audio feature extraction functions parameterized by distinct deep networks. Their specific implementations can correspond to diverse model architectures, including ResNet, YOLOv11, ViT, Swin-T, CNN14, and HTSAT. After feature extraction, the corresponding deep semantic feature representations can be obtained as follows:

(1) $$f_{v}=\phi_{v}\left(x_{v}\right),f_{a}=\phi_{a}\left(x_{a}\right)$$

where $f_{v}$ and $f_{a}$ denote the high-level feature representations of the visual and audio modalities, respectively.

In the classification stage, the model maps the features through a linear classification layer and utilizes the Softmax function to output the category probability distribution:

(2) $$p\left(y|f\right)=\mathrm{Softmax}\left(Wf+b\right)$$

where W and b represent the weight matrix and bias parameter of the classification layer, respectively.

Through the aforementioned feature representation and classification modeling formulation, we can systematically analyze the unimodal learning capabilities of various network architectures on the AVB81 dataset. Furthermore, we evaluate the adaptability and generalization capability of different visual and acoustic features for the fine-grained bird recognition task, thereby comprehensively validating the learnability, stability, and challenge of the AVB81 dataset.

### 4.2. Cross-Paradigm Audiovisual Fusion Framework

To further validate the practical application value and modeling adaptability of the AVB81 dataset in multimodal learning tasks, this paper constructs a cross-paradigm audiovisual fusion evaluation framework. This framework is anchored by three classic levels of multimodal fusion—early fusion, mid-fusion, and late fusion—and introduces fusion strategies encompassing shallow signal coupling, deep semantic interaction, and decision synergy. Consequently, it systematically verifies the multimodal modeling capability of AVB81 in the fine-grained bird audiovisual classification task. This framework not only provides a unified and reproducible experimental benchmark for subsequent multimodal bird recognition research, but also offers crucial data and experimental support for an in-depth exploration of fine-grained audiovisual synergistic modeling mechanisms.

#### 4.2.1. Early Fusion

Early fusion completes the integration of multimodal information directly at the input representation stage. Its core idea is to jointly model the raw visual and audio signals before the network forms high-level semantic representations. Given the significant differences between images and audio spectrograms in terms of statistical distribution, spatial structure, and texture patterns, this fusion paradigm effectively reflects the dataset’s adaptability and information complementarity in the joint representation of low-level heterogeneous modalities. Based on this, we design the following three early fusion strategies to evaluate the application performance of the AVB81 dataset in early audiovisual fusion scenarios.

(1) Channel Concatenation Fusion

As the most direct input-level fusion method, this strategy represents the RGB image as $x_{v}\in R^{H\times W\times 3}$ and converts the Mel-spectrogram into a three-channel representation of the same dimensions $x_{a}\in R^{H\times W\times 3}$. Subsequently, concatenation is performed along the channel dimension to obtain the fused input $x_{\mathrm{fuse}}$:

(3) $$x_{\mathrm{fuse}}=\mathrm{Concat}\left(x_{v},x_{a}\right)$$

While this direct coupling strategy projects spatial pixels and time-frequency bins into a unified input space, these two modalities represent fundamentally distinct physical domains that lack native geometric synchronization. However, standard convolutional networks overcome this structural mismatch through mathematical linear decomposition. Specifically, the output feature map Y generated by a standard 2D convolutional filter $K\in R^{C_{out}\times 6\times k\times k}$ ($C_{out}$ is the number of output channels and k is the kernel size) acting on the 6-channel input can be explicitly formulated as two independent unimodal operations:

(4) $$Y=\sum_{c=1}^{3}K_{c}*X_{fuse,c}^{v}+\sum_{c=4}^{6}K_{c}*X_{fuse,c}^{a}$$

This formulation explicitly demonstrates that, in the initial layer, the convolution effectively executes parallel, domain-specific feature extractions and merges them via element-wise addition, rather than causing chaotic local feature corruption within the kernel’s receptive field. As the network deepens, it does not rely on explicit low-level physical coordinate alignment; instead, it leverages its global capacity to capture high-level statistical co-occurrences between visual phenotypes and acoustic traits. Ultimately, this strategy is utilized to observe the joint representation performance of AVB81 under the direct coupling of low-level heterogeneous modalities, serving as an essential baseline.

(2) Convolutional Feature Mapping Fusion

Considering the obvious differences in statistical distributions and local structural patterns between raw images and spectrograms, we first perform independent local feature encoding on the visual and audio inputs to construct richer shallow modality representations. To this end, independent convolutional projection layers Conv (·)  are utilized to encode the local features of the visual and audio inputs, respectively:

(5) $$E_{v}={\mathrm{Conv}}_{v}\left(x_{v}\right),E_{a}={\mathrm{Conv}}_{a}\left(x_{a}\right)$$

Subsequently, fusion is accomplished at the feature map level:

(6) $$E_{\mathrm{fuse}}=\mathrm{Concat}\left(E_{v},E_{a}\right)$$

The fused joint features $E_{\mathrm{fuse}}$ are further fed into the subsequent network for global relational modeling and semantic representation learning. This method aims to observe the representation of visual local textures and acoustic time-frequency structures at the shallow feature level, as well as the overall performance of AVB81 under local structural feature fusion conditions.

(3) Additive Fusion

Element-wise additive fusion is another prevalent joint representation approach in early multimodal fusion. Under the premise of ensuring identical spatial and channel dimensions for both the image and the spectrogram, element-wise addition is applied to the two modalities to obtain the fused input $x_{\mathrm{fuse}}$:

(7) $$x_{\mathrm{fuse}}=x_{v}+x_{a}$$

Compared with the channel concatenation method, this strategy avoids expanding the feature channel dimension. Instead, it accomplishes the joint representation of visual and audio information through element-wise superposition within a shared space, thereby further highlighting the co-response features of different modalities at corresponding spatial locations. This strategy is primarily employed to observe the information retention and overall fusion performance of the visual and audio modalities in the AVB81 dataset under the condition of direct superposition.

#### 4.2.2. Mid-Fusion

Mid-fusion accomplishes cross-modal information interaction within the deep semantic feature space and is currently the most widely applied fusion paradigm in multimodal learning. Compared to early fusion, which focuses more on low-level signal alignment, mid-fusion places greater emphasis on high-level semantic relational modeling and cross-modal dependency learning. This paradigm typically achieves more stable semantic complementarity and discriminative enhancement while preserving the independent representation capabilities of each modality.

To mitigate the impact of feature distribution discrepancies across different modalities, visual and audio features are first mapped into a unified semantic space via a feature projection layer, followed by various forms of cross-modal fusion operations. Furthermore, existing work, MSFG-AVFNet [4], has preliminarily validated the effectiveness of the mid-fusion strategy based on deep feature space concatenation on the AVB81 dataset. Therefore, in the intermediate fusion experiments, this study adopts the same network architecture as MSFG-AVFNet. Building upon this foundation, we further extend the framework with various cross-modal interaction mechanisms to systematically investigate the impact of different fusion methods on multimodal classification performance in the AVB81.

(1) Concatenation Fusion

As the most fundamental feature-level fusion strategy, this method directly concatenates features along the feature dimension. Let the deep semantic features extracted by the visual and audio encoders (and mapped to the unified space) be respectively denoted as $f_{v}$ and $f_{a}$. The fused joint representation $f_{\mathrm{fuse}}$ is then formulated as:

(8) $$f_{\mathrm{fuse}}=\mathrm{Concat}\left(f_{v},f_{a}\right)$$

By preserving the complete visual and audio semantic features to form a joint representation, this method allows the two modalities to maintain relatively independent feature structures after fusion. It is suitable for observing the complementary relationships and joint expression performance of visual appearance features and vocalization semantic features within the deep semantic space in the AVB81 dataset.

(2) FiLM Fusion

FiLM (Feature-wise Linear Modulation) achieves cross-modal feature interaction via conditional modulation. This method utilizes one modality to generate scaling and shifting parameters, applying a dynamic affine transformation to the other modality:

(9) $$\mathrm{FiLM}\left(f_{v}\right)=\gamma \left(f_{a}\right)⊙f_{v}+\beta \left(f_{a}\right)$$

where γ(⋅) and β(⋅) represent the scaling and shifting functions generated by the conditioning modality, respectively, and ⊙ denotes element-wise multiplication.

By conditionally modulating the features of one modality based on the other, this method endows the fusion process with a certain degree of dynamic adaptability. This strategy is primarily employed to observe the conditional dependency relationships between the visual and audio modalities in the AVB81 dataset, as well as the impact of different modality information on the joint representation.

(3) Gated Fusion

To enhance the adaptive adjustment capability for different modality information during the fusion process, we introduce a dynamic gated fusion mechanism. This mechanism adaptively adjusts the importance weights of different modalities through a learnable gating function:

(10) $$f_{\mathrm{fuse}}=g⊙f_{v}+\left(1-g\right)⊙f_{a}$$

where g denotes the gating weight dynamically learned by the network.

This method can automatically regulate the contribution ratios of visual and audio features based on the modality quality of the current input sample, thereby achieving more stable multimodal synergistic modeling in complex scenarios. This strategy is mainly utilized to observe the fusion performance on the AVB81 dataset under varying modality quality conditions, along with the influence of the dynamic modality selection mechanism on the joint semantic representation.

#### 4.2.3. Late Fusion

Late fusion divides multimodal learning into relatively independent modeling processes, where each modality independently completes feature extraction and classification prediction, ultimately fusing the output results at the decision level. Compared with early fusion and mid-fusion, late fusion does not directly conduct cross-modal feature interaction. Instead, it accomplishes joint decision-making while maintaining the independent prediction results of each modality, thereby effectively preserving the independent representation capabilities of the unimodal branches while inherently mitigating noise propagation through decision-level confidence imbalances.

Let the output probability distributions of the visual and audio classification branches be denoted as $P_{v}$ and $P_{a}$. We design the following three late fusion strategies:

(1) Score Average

This method directly performs an arithmetic average on the Softmax probabilities output by the classification heads of the visual and audio modalities:

(11) $$P_{\mathrm{fuse}}=\frac{1}{2}\left(P_{v}+P_{a}\right)$$

where $P_{\mathrm{fuse}}$ represents the final fused probability distribution.

This strategy directly fuses the prediction results of the visual and audio branches, making it suitable for observing the consistency between the prediction results of different modalities in the AVB81 dataset as well as the fundamental decision fusion performance.

(2) Weighted Fusion

Considering that different bird categories exhibit varying degrees of dependency on visual and acoustic information, adjustable fusion weights are further introduced on the basis of probability average fusion:

(12) $$P_{\mathrm{fuse}}=\alpha P_{v}+\beta P_{a}$$

where α and β represent the fusion weights for the visual and audio branches, respectively.

By adjusting the fusion ratios of different modalities to perform joint decision-making, this method can be utilized to observe the relative contribution changes of the visual and audio modalities at the decision level within the AVB81 dataset.

(3) Logit Fusion

Unlike probability-level fusion, logit fusion accomplishes cross-modal joint modeling prior to Softmax activation. Specifically, the logit representations $z_{v}$ and $z_{a}$ of the visual and audio modalities are first added together:

(13) $$z_{\mathrm{fuse}}=z_{v}+z_{a}$$

where $z_{\mathrm{fuse}}$ is the fused logit representation. Subsequently, the final prediction result is output through the classification layer by applying the Softmax function to $z_{\mathrm{fuse}}$.

Compared with probability distributions, logit representations retain more unnormalized categorical response information. Therefore, this strategy is primarily employed to observe the joint fusion performance of different modalities in the AVB81 dataset under the condition of unnormalized representations at the decision level.

### 4.3. Implementation Details

For the visual modality in videos, Video-Frame, we first uniformly divide each video into 10 non-overlapping temporal segments, each lasting 1 s. Within each segment, one frame is sampled as a representative keyframe, thereby forming a temporal visual sequence of 10 frames. During the feature modeling phase, all visual inputs follow a unified image encoding pipeline, which maps frame-level inputs to the feature representation space. Under different experimental settings, this encoding process can be implemented by various visual backbone networks, while the input preprocessing and output feature interfaces remain consistent. At this stage, the temporal aggregation strategy explicitly diverges based on the fusion paradigm. For mid-fusion and late-fusion architectures, max pooling aggregation is performed on the 10 frame-level features to obtain a video-level visual representation, thereby achieving global modeling of temporal information. However, for early fusion strategies, applying temporal max pooling to shallow visual frames is unfeasible, as it would destroy critical local textures and lead to severe pixel-level aliasing. Therefore, we adopt a temporal jittering strategy: randomly sampling one frame during training as a temporal augmentation, and selecting the center frame during testing as a deterministic anchor.

For the image modality, Birdsnap Image, since it consists of static image data, we directly employ the same visual encoding pipeline as Video-Frame for feature extraction, without involving the temporal sampling and cross-frame feature aggregation processes. Regarding data preprocessing, for the YOLOv11 model, all Video-Frames and their corresponding Birdsnap Images from the same species are uniformly resized to a resolution of 640 × 640 as the network input. For the ResNet50, ViT-B, and Swin-T models, random cropping is applied to generate 224 × 224 local regions as model inputs. All models incorporate data augmentation strategies such as random flipping, random scaling, and perturbations in brightness, saturation, and hue. These strategies are utilized to enhance the models’ adaptability to illumination variations, scale changes, and complex background conditions. During the testing phase, only resizing and normalization are performed.

For the audio modalities Unpaired Audio and Video-Audio, we first uniformly resample the raw audio signals to 16 kHz to ensure sampling consistency. Subsequently, the short-time Fourier transform is applied to map the signals from the time domain to the time-frequency domain, with a window size of 1024 and a hop length of 320. The resulting spectrograms are further mapped to 64 Mel filter banks (50 Hz–8 kHz), followed by logarithmic magnitude transformation and normalization, generating standard Mel-spectrogram representations. For a 10 s audio clip, the corresponding spectrogram size is approximately 64 × 501. During the feature modeling phase, all audio inputs undergo representation learning via a unified acoustic encoder. Different models vary only in their encoder architectures, while the input feature format remains identical. Time masking and frequency masking augmentation strategies are employed during the training process to enhance the model’s robustness against complex acoustic environments.

All experiments in this paper are implemented based on the PyTorch framework. The hardware and software environment configurations for the experiments are detailed in Table 2.

During the model training process, with the exception of YOLOv11 which employs the SGD optimizer, all other models utilize the Adam optimizer for parameter optimization. For YOLOv11 on the Birdsnap Image, the initial learning rate is set to $1\times 10^{-2}$; while for ViT-B, it is set to $1\times 10^{-5}$. The initial learning rates for all other models and configurations are uniformly set to $1\times 10^{-4}$. The batch size for all experiments is set to 32. In the unimodal experiments, the number of training epochs for YOLOv11 on Birdsnap Image is set to 500, while for the other models on Birdsnap Image and Unpaired Audio, it is uniformly set to 200 epochs. For Video-Frame and Video-Audio, the training epochs for all models are uniformly set to 30. For the multimodal fusion experiments, the training epochs for all fusion models are uniformly set to 100. During the training process, cross-entropy loss is utilized as the loss function, and the model achieving the optimal performance across all experiments is saved as the final model for evaluation.

To evaluate the classification performance on the AVB81 dataset under different models and fusion strategies, this paper adopts Accuracy and F1-score as quantitative evaluation metrics. Specifically, Accuracy is utilized to measure the overall classification correctness of the model; the F1-score comprehensively considers Precision and Recall, enabling it to more thoroughly reflect the overall classification performance and category discrimination stability of the model in the fine-grained bird classification task. To ensure empirical stability and mitigate stochastic fluctuations, all experiments are conducted over five independent runs using distinct random seeds (42, 100, 2026, 777, and 999). Accordingly, the evaluation metrics are reported as Mean ± Standard Deviation (mean ± std).

## 5. Experiments and Results

### 5.1. Unimodal Benchmark Evaluation

To systematically analyze the feature distributions and classification difficulties of the AVB81 dataset under different modalities and data presentation formats, this study constructs unimodal benchmark experiments on the visual and audio modalities, respectively. Considering that video data exhibits higher complexity in terms of background variations, target scales, and environmental noise, for the evaluation of Video-Frame and Video-Audio, we conduct experiments referring to the multi-stage fine-tuning paradigm of MSFG-AVFNet. Specifically, the first stage is the multimodal fine-tuning stage: the encoders pre-trained on ImageNet are first fine-tuned on high-quality single-modal data (Birdsnap Image and Unpaired Audio) to learn more stable visual and acoustic feature representations of birds. The second stage is the video-enhanced fine-tuning stage: the encoders trained in the first stage are further fine-tuned on the video target-domain data to enhance the models’ adaptability to spatial-temporal variations and environmental interferences in video scenarios. Crucially, since the multimodal fine-tuning stage aims to establish a deterministic and controlled checkpoint for downstream video-level learning, the evaluation outcomes at this stage are naturally deterministic and bypass multi-seed random testing. Consequently, the performance metrics corresponding to the multimodal fine-tuning stage are reported without mean and standard deviations, whereas the full unimodal classification results across multiple seeds are detailed separately for the visual and audio modalities.

In the visual modality evaluation, the AVB81 dataset exhibits obvious cross-domain disparities between the Birdsnap Image and Video-Frame scenarios. As shown in Table 3, all models achieve relatively strong classification performance on the static Birdsnap Image, with Transformer architectures outperforming CNN architectures overall. Swin-T achieves the highest Accuracy of 81.54% and F1-score of 81.67% on Birdsnap Image, indicating that the hierarchical attention mechanism possesses superior modeling capability for the fine-grained local features of birds. ViT-B similarly demonstrates high performance. However, as shown in Table 4, when the models are transferred from Birdsnap Image to Video-Frame data, a significant performance degradation is observed across all architectures. ResNet-50 drops from 73.48% to 61.47% Accuracy, while YOLOv11 exhibits the largest decline, decreasing from 75.64% to 53.15%. This phenomenon indicates that motion blur, background interference, target scale variations, and complex illumination conditions in video scenarios significantly exacerbate the difficulty of fine-grained visual recognition. Although Transformer-based models also suffer performance degradation, they maintain superior performance compared with CNN-based methods. In particular, ViT-B achieves the highest Accuracy of 69.84% and F1-score of 69.73% on Video-Frame data, demonstrating the stronger global feature modeling capability and certain robustness of Transformer architectures in complex scenarios.

Compared to the visual modality, the audio modality experiments more intuitively reflect the challenging nature of the AVB81 dataset under authentic wild soundscape conditions. As shown in Table 5, all models achieve relatively satisfactory performance on the independently collected Unpaired Audio dataset. Among them, CNN14, pre-trained on AudioSet, achieves the highest Accuracy of 67.55% and F1-score of 66.30%, while HTSAT also attains an Accuracy of 66.93%. This indicates that under relatively clean audio conditions, bird acoustic features possess good separability. However, as shown in Table 6, when the models are further applied to the Video-Audio data, the performance of all models experiences a significant degradation, with Accuracy generally plummeting to the 14–17% range. For instance, CNN14 drops from 67.55% to 15.80%. This result demonstrates that, compared to independently collected high-quality Unpaired Audio, Video-Audio contains substantial environmental noise, background sounds, and multi-target overlapping phenomena. These factors cause severe interference with the acoustic features of the target birds, thereby significantly increasing the difficulty of acoustic recognition. In contrast, although Video-Frame is similarly affected by complex environmental factors, it overall maintains a relatively higher classification performance. This suggests that in authentic wild video monitoring scenarios, visual information remains the primary effective information source for current fine-grained bird recognition.

Overall, the unimodal benchmark experiments systematically reveal that the AVB81 dataset simultaneously encompasses two types of feature distributions: high-quality static data and complex video scenarios. Among them, the extremely low classification accuracy exhibited by Video-Audio further illustrates that in real-world wild fine-grained bird video monitoring scenarios, relying solely on a single modality—especially the single audio modality—makes it difficult to obtain stable and reliable recognition results. This further highlights the necessity of conducting research on audiovisual multimodal fusion.

### 5.2. Multimodal Fusion Benchmark Evaluation

#### 5.2.1. Quantitative Performance Analysis

After completing the analysis of the unimodal experiments, this paper further evaluates the performance of different audiovisual fusion strategies on the AVB81 dataset, aiming to systematically verify the complementarity between the visual and audio modalities as well as the dataset’s adaptability to various fusion paradigms. All multimodal fusion experiments are conducted under this weakly aligned multimodal learning setting, and exclusively use synchronized Video-Frame and Video-Audio pairs extracted from the same field recordings for evaluation. In the unimodal experiments, Transformer architectures overall demonstrated stronger visual feature modeling capabilities. Specifically, ViT-B achieved relatively stable classification performance in both the Birdsnap Image and Video-Frame scenarios. Therefore, in subsequent experiments, we adopt ViT-B as the primary visual encoder to mitigate the impact of visual backbone variations on the fusion experiment results, thereby allowing for a more objective analysis of the performance changes induced by the different fusion strategies themselves. Similarly, for the audio modality, ResNet-18 achieved the highest classification performance on the challenging Video-Audio benchmark among the evaluated audio encoders. Therefore, ResNet-18 was adopted as the primary audio encoder in the subsequent multimodal experiments.

Furthermore, for the weighted fusion strategy within the late fusion paradigm, based on the significant performance disparity observed between the reliable visual modality and the noise-prone acoustic modality in the aforementioned unimodal tests, we adopt α = 0.8 and β = 0.2 as an initial empirical setting. This setup aims to emphasize the dominant visual cues while preserving supplementary acoustic information. It is worth noting that this is a preliminary baseline setting rather than the absolute optimal combination. Table 7 presents the experimental results under the three typical fusion paradigms: early fusion, mid-fusion, and late fusion.

As shown in Table 7, distinct performance disparities are observed among the different fusion levels. Notably, mid-fusion exhibits robust baseline performance, whereas early fusion and certain late fusion methods demonstrate relatively limited capabilities. This indicates that for audiovisual datasets like AVB81, which simultaneously encompass complex background interferences and significant modality heterogeneity, the level at which fusion occurs and the cross-modal information interaction paradigm exert a pronounced impact on the final classification performance.

In the early fusion stage, the accuracy of all three fusion strategies remains constrained between 51% and 53%. This implies that when directly combining RGB images with Mel-spectrograms, it is challenging for the model to establish stable cross-modal associative relationships within the shallow representation space. Since input-level fusion must simultaneously tackle modality alignment and semantic modeling, rendering the overall optimization relatively difficult. Furthermore, shallow fusion is highly sensitive to noise and local interference, which further compromises the efficacy of joint representation learning.

Compared with early fusion, mid-fusion demonstrates a more stable performance advantage. Under the ViT-B + ResNet-18 configuration, all interaction methods consistently achieve an Accuracy and F1-score of approximately 74%. This confirms that cross-modal joint modeling within the deep semantic space is more conducive to exploiting the complementary information between vision and audio. Compared with directly processing raw heterogeneous inputs, mid-fusion first extracts high-level semantic features via independent encoders before performing cross-modal fusion, thereby mitigating the impact of low-level noise and modality distribution discrepancies on the fusion process to a certain extent. Notably, significant performance differences are observed among different backbone network architectures. The intermediate fusion model based on YOLOv11 and CNN14 achieves an Accuracy of only 57.07% under the Concatenation Fusion. This indicates that the representation capacity of individual single-modal backbones strictly bounds the downstream cross-modal synergy.

In the late fusion stage, distinct performance disparities are exhibited among different fusion strategies. Notably, Weighted Fusion achieves a highly competitive Accuracy of 74.82% and an F1-score of 74.69%. In contrast, Score Average merely attains an Accuracy of 72.15%, and Logit Fusion further drops to 70.42%. This result indicates that the contributions of different modalities to the final classification task are not entirely consistent. By introducing fusion weights, Weighted Fusion can adjust the contribution ratios of the visual and audio branches, thereby stably utilizing the complementary information between different modalities. In comparison, Score Average directly averages the predicted probabilities without considering potential quality discrepancies between different modalities; meanwhile, Logit Fusion performs fusion directly within the unnormalized decision space, rendering its joint decision-making outcomes relatively limited.

#### 5.2.2. Exploring the Optimization Potential of Mid-Fusion

The true advantage of the mid-fusion architecture lies in its exceptional extensibility and capacity to integrate deep multi-modal training constraints. To investigate this potential, we introduce the advanced audiovisual loss function featuring intra-modal stability and inter-modal consistency constraints from MSFG-AVFNet into the mid-fusion baseline model, which adaptively suppresses noise propagation through intra-modal stability and inter-modal consistency constraints when the other modality undergoes quality degradation. The experimental results are presented in Table 8.

As indicated in Table 7 and Table 8, migrating from the standard cross-entropy loss function to the advanced audiovisual loss optimization delivers a consistent and significant performance enhancement across all backbone architectures. For the YOLOv11 + CNN14 configuration, the multi-modal joint loss function drives a substantial performance leap of up to 7.53%, elevating the recognition accuracy from 57.07% to 64.60%. This significant improvement demonstrates that even when the representation capacity of the backbone network is limited, a specialized multi-modal loss function can successfully guide the mid-fusion layer to suppress noise propagation. For the ViT-B + ResNet-18 configuration, the multi-modal loss function enables the accuracies of all three cross-modal interaction strategies to exceed 76%. Notably, Gated Fusion combined with the audiovisual loss function establishes the optimal benchmark for the entire dataset, achieving an accuracy of 76.18% and an F1-score of 75.99%. This systemic enhancement demonstrates that mid-fusion is not a rigid feature combination process; rather, its architectural space possesses unique optimization flexibility.

#### 5.2.3. Statistical Significance Analysis

To mathematically validate that the performance divergence among these fusion strategies is statistically significant rather than being an accidental phenomenon caused by random variance, a standard paired two-sample *t*-test was performed on the 5-seed accuracy matrices, and the results are presented in Table 9.

The statistical outcomes in Table 9 indicate that under the uniform cross-entropy baseline, FiLM Fusion significantly outperforms all early fusion strategies, while also yielding statistical advantages over decision-level methods such as Score Average and Logit Fusion, with all corresponding *p*-values staying well below the 0.05 threshold. However, its performance difference compared to Weighted Fusion is not statistically significant (*p* = 0.06346241), which reveals that without specialized optimization constraints, pure deep feature interaction achieves a performance bound comparable to a decision-level prior that relies on fixed manual weights to avoid acoustic noise. In contrast, when integrating specialized multi-modal training constraints, Gated Fusion (audiovisual loss) achieves a comprehensive and statistically significant advantage over every single alternative configuration (*p* < 0.05). Most notably, when evaluated directly against the competitive Weighted Fusion baseline, the resulting *p*-value drops to 0.00011809, which falls substantially below the standard significance threshold. This mathematical confirmation demonstrates that executing cross-modal alignment within a deep semantic feature space, paired with specialized joint-loss optimization, provides a robust and adaptive representation advantage for fine-grained bird recognition in complex wild scenarios.

In summary, the experimental results demonstrate that the AVB81 dataset can effectively support diverse typical audiovisual fusion paradigms and clearly reflect the performance disparities among different fusion mechanisms, thereby highlighting the research value and application potential of the AVB81 dataset in complex cross-modal learning tasks. Among them, mid-fusion based on the deep semantic space exhibits the optimal overall performance. This indicates that the fine-grained bird recognition task relies more heavily on high-level semantic complementary information, rather than merely on shallow input-level coupling.

## 6. Conclusions

This paper constructs and systematically evaluates AVB81, a multimodal dataset tailored for fine-grained bird video recognition. Encompassing diverse formats—static images (Birdsnap Image), independent audio (Unpaired Audio), as well as Video-Frame and Video-Audio extracted from videos—AVB81 authentically reflects the complex environmental interferences and cross-modal learning challenges inherent in wild scenarios. Through comprehensive unimodal and multimodal benchmark experiments, we demonstrate the performance disparities among different network architectures and fusion paradigms. Ultimately, AVB81 provides a novel data foundation and robust experimental basis for cross-modal semantic modeling and fine-grained recognition in complex natural scenes. Beyond serving as an algorithmic benchmark, this multimodal framework holds significant practical value for biological research. Traditional automated wildlife surveys and passive acoustic monitoring systems often struggle with severe habitat complexities, such as long-distance visual occlusions and environmental biophonic. By validating the complementarity of visual and acoustic information, our work paves the way for deploying intelligent monitoring systems on edge-AI devices across nature reserves. Such multi-dimensional perception capabilities can effectively process asynchronous multimedia streams, reducing reliance on manual observation and directly supporting large-scale ecological assessments and biodiversity conservation strategies.

Despite these contributions, we explicitly acknowledge that AVB81 inherently possesses limitations. First, regarding geographical bias and taxonomic imbalance, the dataset focuses specifically on 81 North American bird species. Interestingly, while the static image modality was strictly curated to maintain class balance (approximately 60 training samples per species), the audio and video modalities inevitably exhibit varying degrees of sample imbalance (e.g., audio training samples range from 7 to 280) due to the varying availability of wild observations. Second, regarding recording quality variance and environmental noise, while AVB81 successfully captures the authentic complexities of wild habitats, the limitation lies in the absence of detailed environmental labels. In the real world, multiple interferences (e.g., wind noise, motion blur, and foliage occlusion) often occur simultaneously within a single sample. Because we cannot decouple these naturally mixed noises, it is difficult to test a model’s robustness against one specific type of interference independently. Finally, integrating independent repositories inevitably leads to source heterogeneity, as these distinct repositories inherently contain varying recording formats and introduce subtle cross-domain shifts that current models must overcome. Methodologically, as current experiments primarily focus on supervised classification, there is ample room to explore weakly supervised, self-supervised, and large-scale pre-training-driven cross-modal learning paradigms. Second, our future research will expand the application scope of AVB81 to cover more complex tasks, such as cross-modal retrieval, temporal behavioral analysis, and fine-grained evaluations across specific environmental subsets (e.g., silent vs. actively vocalizing videos), to meet the demands of wild ecological monitoring.

## Figures and Tables

**Table 1:** Comprehensive comparison of AVB81 with existing bird-related datasets.

Dataset	Species Quantity	Modalities	Sample Size	Alignment Level	Benchmark Availability
CUB-200-2011 [5]	200	Image	11,788 images	N/A	Multi-class classification and part localization
iNat2021 [16]	1486	Image	429,707 images	N/A	Fine-grained bird visual classification
NABirds [6]	555	Image	48,562 images	N/A	Fine-grained bird visual classification
Birds-525 [17]	525	Image	89,885 images	N/A	Fine-grained bird visual classification
DB3V [8]	10	Audio	+25 h of audio recordings	N/A	Cross-corpus bird species recognition
Birdset [7]	10,000	Audio	+6800 h of audio recordings	N/A	Multi-label sound classification, covariate shift, or self-supervised learning
BirdCLEF2023 [18]	264	Audio	16,900 audios	N/A	bird sound classification
Xeno-canto [14]	10,563	Audio	+1,000,000	N/A	Bird sound classification
IBC127 [11]	127	Video (Visual-only)	8014 videos	N/A	Fine-grained bird video classification
FGB-AV [12]	45	Video	10,495 videos	Sample-level	Weakly supervised fine-grained video recognition
WetlandBirds [19]	13	Video (Visual-only)	178 videos	N/A	Bird species identification and behavior recognition
VB100 [9]	100	Video, Audio	1416 videos, 502 audios	Hybrid (Sample and Species)	Fine-grained object classification (Visual-only)
YouTube-Birds [10]	200	Video (Visual-only), Image	18,350 videos, 11,788 images	Species-level	Fine-grained bird video classification
SSW60 [13]	60	Video, Image, Audio	5400 videos, 31,221 images, 3861 audios	Hybrid (Sample and Species)	Multimodal Fusion and Learning
AVB81 (Ours)	81	Video, Image, Audio	3247 videos, 7083 images, 5418 audios	Hybrid (Sample and Species)	Multimodal Fusion and Learning

**Table 2:** The settings of experiments.

Test Environment	Type
System	Ubuntu 20.04.1 LTS
Graphics card	NVIDIA GeForce RTX 3080
CPU	AMD Ryzen 9 5900X @4.8Ghz
RAM	128 G
Programming language	Python 3.10

**Table 3:** Visual unimodal classification results in the multimodal fine-tuning stage.

**Model Category**	**Model**	**Visual** **Modality**	**Pre-Training Dataset**	**Accuracy (%)**	**F1-Score (%)**
CNN-based	ResNet-50	Birdsnap Image	ImageNet	73.48	73.52
CNN-based	YOLOv11	Birdsnap Image	ImageNet	75.64	75.57
Transformer-based	ViT-B	Birdsnap Image	ImageNet	79.93	79.98
Transformer-based	Swin-T	Birdsnap Image	ImageNet	81.54	81.67

**Table 4:** Visual unimodal classification results in the video-enhanced fine-tuning stage.

Model Category	Model	Visual Modality	Pre-Training Dataset	Accuracy (Mean ± Std) (%)	F1-Score (Mean ± Std) (%)
CNN-based	ResNet-50	Video-Frame	Birdsnap Image	61.47 ± 1.01	61.34 ± 0.90
CNN-based	YOLOv11	Video-Frame	Birdsnap Image	53.15 ± 0.14	53.12 ± 0.14
Transformer-based	ViT-B	Video-Frame	Birdsnap Image	69.84 ± 0.48	69.73 ± 0.50
Transformer-based	Swin-T	Video-Frame	Birdsnap Image	67.84 ± 0.83	67.79 ± 0.87

**Table 5:** Audio unimodal classification results in the multimodal fine-tuning stage.

**Model Category**	**Model**	**Audio** **Modality**	**Pre-Training** **Dataset**	**Accuracy (%)**	**F1-Score (%)**
CNN-based	ResNet-18	Unpaired Audio	ImageNet	62.63	61.01
CNN-based	CNN14	Unpaired Audio	AudioSet	67.55	66.30
Transformer-based	ViT-B	Unpaired Audio	ImageNet	58.39	57.36
Transformer-based	HTSAT	Unpaired Audio	AudioSet	66.93	65.67

**Table 6:** Audio unimodal classification results in the video-enhanced fine-tuning stage.

Model Category	Model	Audio Modality	Pre-Training Dataset	Accuracy (Mean ± Std) (%)	F1-Score (Mean ± Std) (%)
CNN-based	ResNet-18	Video-Audio	Unpaired Audio	16.08 ± 0.46	15.79 ± 0.57
CNN-based	CNN14	Video-Audio	Unpaired Audio	15.80 ± 0.46	14.74 ± 0.65
Transformer-based	ViT-B	Video-Audio	Unpaired Audio	14.81 ± 0.56	14.07 ± 0.68
Transformer-based	HTSAT	Video-Audio	Unpaired Audio	15.50 ± 0.41	14.75 ± 0.72

**Table 7:** Audiovisual multimodal fusion classification results.

Fusion Strategy	Fusion Level	Model	Pre-Training Dataset	Accuracy (Mean ± Std) (%)	F1-Score (Mean ± Std) (%)
Channel Concatenation Fusion	Early	ViT-B	ImageNet	52.44 ± 2.16	51.63 ± 2.28
Convolutional Feature Mapping Fusion	Early	ViT-B	ImageNet	51.39 ± 1.07	50.74 ± 0.92
Additive Fusion	Early	ViT-B	ImageNet	52.02 ± 0.45	51.28 ± 0.76
Concatenation Fusion	Middle	YOLOv11 + CNN14	Birdsnap Image+ Unpaired Audio	57.07 ± 0.59	57.16 ± 0.64
Concatenation Fusion	Middle	ViT-B + ResNet-18	Birdsnap Image+ Unpaired Audio	73.99 ± 0.36	73.72 ± 0.38
FiLM Fusion	Middle	ViT-B + ResNet-18	Birdsnap Image+ Unpaired Audio	74.04 ± 0.41	73.73 ± 0.41
Gated Fusion	Middle	ViT-B + ResNet-18	Birdsnap Image+ Unpaired Audio	74.00 ± 0.36	73.69 ± 0.40
Score Average	Late	ViT-B + ResNet-18	Birdsnap Image+ Unpaired Audio	72.15 ± 0.70	71.74 ± 0.67
Weighted Fusion	Late	ViT-B + ResNet-18	Birdsnap Image+ Unpaired Audio	74.82 ± 0.81	74.69 ± 0.76
Logit Fusion	Late	ViT-B + ResNet-18	Birdsnap Image+ Unpaired Audio	70.42 ± 0.93	69.93 ± 0.88

**Table 8:** Audiovisual multimodal fusion classification results with audiovisual loss optimization.

Fusion Strategy	Model	Pre-Training Dataset	Accuracy (Mean ± Std) (%)	F1-Score (Mean ± Std) (%)
Concatenation Fusion	YOLOv11 + CNN14	Birdsnap Image+ Unpaired Audio	64.60 ± 0.36	64.42 ± 0.38
Concatenation Fusion	ViT-B + ResNet-18	Birdsnap Image+ Unpaired Audio	76.03 ± 0.69	75.87 ± 0.75
FiLM Fusion	ViT-B + ResNet-18	Birdsnap Image+ Unpaired Audio	76.15 ± 0.72	75.96 ± 0.80
Gated Fusion	ViT-B + ResNet-18	Birdsnap Image+ Unpaired Audio	76.18 ± 0.65	75.99 ± 0.73

**Table 9:** Paired *t*-test results comparing alternative fusion paradigms.

Model A	Model B	*p*-Value
FiLM Fusion (cross-entropy loss)	Channel Concatenation Fusion	0.00003926
	Convolutional Feature Mapping Fusion	0.00000567
	Additive Fusion	0.00000001
	Score Average	0.00233237
	Weighted Fusion	0.06346241
	Logit Fusion	0.00105660
Gated Fusion (audiovisual loss)	Channel Concatenation Fusion	0.00002980
	Convolutional Feature Mapping Fusion	0.00000662
	Additive Fusion	0.00000024
	Score Average	0.00019831
	Weighted Fusion	0.00011809
	Logit Fusion	0.00033338

## Data Availability

The original data presented in the study are openly available in https://doi.org/10.5281/zenodo.20646496.

## Abbreviations

The following abbreviations are used in this manuscript:

CNN	Convolutional Neural Network
ResNet	Residual Network
ViT-B	Vision Transformer Base
Swin-T	Hierarchical vision transformer using shifted windows
HTSAT	Hierarchical Token-Semantic Audio Transformer
MSFG-AVFNet	Multi-Stage Fine-Grained Audiovisual Fusion Network
FiLM	Feature-wise Linear Modulation
Concat	Concatenate

## Appendix A

The macro-level geographic and habitat breakdown data for the 81 bird species in the AVB81 dataset are shown in Table A1.

**Table A1:** Macro-level geographic and habitat breakdown information on 81 bird species. * indicates subspecies.

Bird Species ID	Scientific Name	Geographic Distribution	Habitat
0	*Sitta pygmaea*	* *melanotis* (Van Rossem, 1929)—extreme SW Canada (S British Columbia), and mountains of W USA (Cascades, Sierra Nevada, Rocky Mts and desert ranges of Great Basin) S to California (Riverside County) and E to W Montana, SW South Dakota (Black Hills), W Nebraska (Pine Ridge area), NW Colorado (Douglas Mts), New Mexico and W Texas (Davis Mts, Guadalupe Mts), also extreme N Mexico in NE Sonora (San Jose Mts) and NW Coahuila (Sierra del Carmen). * *pygmaea* (Vigors, 1839)—C coastal California (from Mendocino County S to San Luis Obispo County). * *leuconucha* (Anthony, 1889) -Southwestern California coast.	Forests
1	*Piranga ludoviciana*	Breeds S Alaska, and W Canada (from British Columbia and S Mackenzie, N Alberta and S Saskatchewan) S in W USA (E to Wyoming and C Colorado) to SW California, S Arizona, New Mexico and W Texas, and extreme NW Mexico (N Baja California). Migrates to mountains of W Mexico (from Jalisco and S Tamaulipas) S locally to Costa Rica and, rarely, W Panama.	Forests
2	*Buteo jamaicensis*	*alascensis* (Grinnell, 1909)—SE Alaska (USA) and coastal British Columbia (W Canada). *harlani* (Audubon, 1830)—interior of Alaska, SW Yukon and N British Columbia. *calurus* (Cassin, 1856)—W North America W of Great Plains.	Grasslands
3	*Calidris alba*	Canadian Arctic, Greenland and Svalbard to Severnaya Zemlya Is, Taymyr Peninsula, Lena Delta and New Siberian Is; small numbers in N Alaska. Winters on coast from C North to S South America, and from W & S Europe and Africa through S Asia to Australasia and some tropical Pacific islands.	Shorelines
4	*Calidris virgata*	C & S Alaska and C Yukon. Winters on Pacific coast of America, from Alexander Archipelago (SE Alaska) S to Straits of Magellan.	Shorelines
5	*Calypte costae*	SW & WC USA (from C California, S Nevada and extreme SW Utah) S to NW Mexico (to S Baja California and C Sonora). Winters S to WC Mexico (at least to Nayarit).	Deserts
6	*Nycticorax nycticorax*	*nycticorax* (Linnaeus, 1758)—C and S Europe eastwards to C and S Asia, extending N to Japan and S to Timor; Africa and Madagascar. *hoactli* (Gmelin, 1789)—North, Central and South America from S Canada to N Chile and N Argentina. *obscurus* (Bonaparte, 1855)—N Chile and NC Argentina S to Tierra del Fuego. *falklandicus* (Hartert, 1914)—Falkland Is.s	Marshes
7	*Lophodytes cucullatus*	SE Alaska S to Oregon; SE Canada S to Mississippi valley.	Lakes and Ponds
8	*Melanerpes formicivorus*	*bairdi* (Ridgway, 1881)—NW Oregon S to N Baja California. *formicivorus* (Swainson, 1827)—Arizona, New Mexico and W Texas S to SE Mexico (W of Chiapas). *angustifrons* (Baird, 1870)—extreme S Baja California.	Open Woodlands
9	*Nannopterum auritum*	*cincinatus* (Brandt, 1837)—Aleutian Is to Alexander Archipelago (W Canada). *albociliatus* (Ridgway, 1884)—Vancouver I to Gulf of California. *auritus* (Lesson, 1831)—Gulf of St Lawrence to Cape Cod and W to Utah. *floridanus* (Audubon, 1835)—North Carolina and Florida S to Cuba.	Lakes and Ponds
10	*Tringa flavipes*	Alaska to SC Canada, E to James Bay. Winters from S USA through Mexico, Central America and West Indies to South America, S to Tierra del Fuego.	Marshes
11	*Ardea herodias*	*fannini* (Chapman, 1901)—SE Alaska to coastal Washington. *herodias* (Linnaeus, 1758)—most of N & C America. *wardi* (Ridgway, 1882)—Kansas and Oklahoma to Florida.	Marshes
12	*Numenius phaeopus*	*phaeopus* (Linnaeus, 1758)—Iceland, Faeroes and N Scotland through Scandinavia to R Yenisey and SW Taymyr; winters from extreme SW Europe and Africa through Middle East to W India, Sri Lanka and Andaman and Nicobar Is. *alboaxillaris* (Lowe, 1921)—steppes N of Caspian Sea; winters on islands and coasts of W Indian Ocean. *variegatus* (Scopoli, 1786)—NE Siberia, from Verkhoyansk Mts, and perhaps SE Taymyr, to basins of R Kolyma and R Anadyr; winters from E India to Taiwan, and S through Philippines and Indonesia to Australasia. *hudsonicus* (Latham, 1790)—W & N Alaska, E to W Yukon and NW Mackenzie, and also W Hudson Bay; winters from S USA to S South America.	Shorelines
13	*Sayornis nigricans*	*semiatra* (Vigors, 1839)—W USA (S from coastal Oregon, SE to W & C Texas) and W Mexico (Baja California, and S in interior to Isthmus of Tehuantepec). *nigricans* (Swainson, 1827)—NE, C & S Mexico. *aquatica* (P. L. Sclater & Salvin, 1859)—Mexico (S of Isthmus of Tehuantepec) S to C Honduras and NW Nicaragua.	Open Woodlands
14	*Egretta thula*	* *brewsteri* (Thayer & Bangs, 1909)—W USA, Baja California. * *thula* (Molina, 1782)—North, Central and South America: from NE USA through Caribbean to NE Argentina; and from NW Mexico to S Chile.	Marshes
15	*Charadrius vociferus*	* *vociferus* (Linnaeus, 1758)—Canada, USA and Mexico; winters S to NW South America. * *ternominatus* (Bangs & Kennard, 1920)—Greater Antilles. * *peruvianus* (Chapman, 1920)—Peru and NW Chile.	Grasslands
16	*Mareca penelope*	Iceland, N Europe, N Asia.s	Lakes and Ponds
17	*Pheucticus ludovicianus*	Breeds S Canada from extreme S District of MacKenzie E to Great Slave L and NE British Columbia, and C & N Alberta, E to Ontario (S from N shore of L Superior), S Quebec (S of c. 49°) and SE Newfoundland, S in USA to NE North Dakota, E South Dakota, NE Nebraska, NC Ohio, Pennsylvania and New Jersey, and inland at higher altitudes to extreme N Georgia. Migrates to region from NE & W Mexico (from SW Tamaulipas and Nayarit) S through Central America to Colombia, Ecuador, Peru, rarely E to Guyana; also West Indies (mostly Bahamas, Cuba, Cayman Is) and sparingly Bermuda.	Forests
18	*Mergus serrator*	Most of N North America, S to Great Lakes; Greenland, Iceland and most of N Eurasia, S to Britain, NE China and N Japan.	Lakes and Ponds
19	*Thalasseus maximus*	*albididorsalis* (Hartert, 1921)—Mauritania to Guinea, occasionally further S; winters S to Namibia. *maximus* (Boddaert, 1783)—S California to Sinaloa, and Maryland (rarely New Jersey) to Texas, and through West Indies to the Guianas and possibly Brazil, with disjunct breeding populations on Yucatán, and in S Brazil, Uruguay and N Patagonia; winters S to Peru and to Uruguay and Argentina.	Shorelines
20	*Arenaria interpres*	*interpres* (Linnaeus, 1758)—Axel Heiberg I and Ellesmere I (N Canadian Arctic), Greenland, N Eurasia and NW Alaska; winters on coasts of W Europe, Africa, S Asia, Australasia and S Pacific islands, with some also on Pacific coast of North America, from California to at least Mexico. *morinella* (Linnaeus, 1766)—NE Alaska and most of Arctic Canada; winters from South Carolina and Gulf of Mexico to SC Chile and N Argentina.	Shorelines
21	*Patagioenas fasciata*	*fasciata* (Say, 1823)—SW British Columbia (including Vancouver I) S through W USA (Washington, California, New Mexico, W Texas) and most of Mexico to Guatemala, Honduras and NC Nicaragua. *vioscae* (Brewster, 1888)—mountains of extreme S tip of Baja California. *letonai* (Dickey & van Rossem, 1926)—border of Honduras and El Salvador, including Volcán de San Miguel.	Forests
22	*Recurvirostra americana*	SE British Columbia E to SW Ontario, and S to N Baja California E to C Texas; E USA; C Mexico. Winters from California and S Texas through Mexico to Guatemala and irregularly to N Honduras; SE USA and Bahamas to Cuba.	Marshes
23	*Rynchops niger*	*niger* (Linnaeus, 1758)—coasts of USA (S California; Massachusetts to Texas) and Mexico (Sonora to Nayarit; Tamaulipas to Yucatán Peninsula); winters from California and North Carolina S to both coasts of Panama. *cinerascens* (Spix, 1825)—coasts of Colombia to mouth of R Amazon and W Ecuador (Gulf of Guayaquil), and large river systems (especially Orinoco and Amazon) S to Bolivia and NW Argentina; winters on coasts, from Ecuador to S Chile, and from Panama to Trinidad and NC Brazil. *intercedens* (Saunders, 1895)—large rivers of E Brazil (W to Maranhão and E Mato Grosso), E Paraguay, Uruguay and NE Argentina (S to Bahía Blanca); winters mainly on coast.	Shorelines
24	*Limnodromus griseus*	*caurinus* (Pitelka, 1950)—S Alaska and S Yukon; winters on Pacific coast from C USA to S Peru. *Hendersoni* (Rowan, 1932)—EC British Columbia to SE Keewatin and C Manitoba; winters from SE USA to Panama. *griseus* (Gmelin, 1789)—C Quebec and W Labrador; winters on Atlantic coast from S USA to Brazil.	Marshes
25	*Aythya collaris*	C Alaska and C Canada eastwards to Newfoundland, southwards to C and W USA, as far S as N California, Colorado and Great Lakes region.	Lakes and Ponds
26	*Podilymbus podiceps*	antillarum Bangs, 1913—Greater and Lesser Antilles. *podiceps* (Linnaeus, 1758)—S & C Canada S to Panama; winters S USA, C America, West Indies. *antarcticus* (Lesson, 1842)—S America, from E Panama S to SC Chile and SC Argentina.	Lakes and Ponds
27	*Icterus cucullatus*	*nelsoni* (Ridgway, 1885)—breeds in SW USA (N California E to W Texas), S to NW Mexico (N Baja California, Sonora and N Chihuahua); non-breeding range extends S to Sinaloa (W Mexico). *trochiloides* (Grinnell, 1927)—C & S Baja California (from San Ignacio S to Cabo San Lucas). *cucullatus* (Swainson, 1827)—breeds from S USA (S Texas in middle Rio Grande Valley) S along edge of Mexican Plateau (Chihuahua and Nuevo León) to Puebla, Oaxaca and S Veracruz; winters from Jalisco S to Guerrero and S Oaxaca, in S Mexico.	Open Woodlands
28	*Egretta tricolor*	*ruficollis* (Gosse, 1847)—S & E USA to C America, W Indies, Colombia and NW Venezuela. *tricolor* (P. L. S. Müller, 1776)—NE Venezuela and Trinidad to NE Brazil; Ecuador and extreme N Peru.	Marshes
29	*Melospiza melodia*	*maxima* Gabrielson & Lincoln, 1951—W Aleutian Is (Attu I E to Atka I). *sanaka* (McGregor, 1901)—E Aleutian Is (Seguam I E to Unimak I and Amak I), Alaska Peninsula E to Stepovak Bay and islands S of peninsula (Sanak I E to Semidi Is). *insignis* (S. F. Baird, 1869)—Kodiak I and Alaska Peninsula at Kukak and Katmai; non-breeding also S along Alaskan coast.	Open Woodlands
30	*Porzana carolina*	SE Alaska, Canada and USA. Winters from S USA, Mexico and West Indies S to C Peru and E to the Guianas.	Marshes
31	*Haemorhous cassinii*	SW Canada (S British Columbia and extreme SW Alberta) and W USA S to extreme NW Mexico (N Baja California); winters S to C Mexico (Coahuila S to Michoacán).	Forests
32	*Leuconotopicus villosus*	* *septentrionalis* (Nuttall, 1840)—from tree-line in Alaska E across S Canada to Ontario, and S to SC British Columbia, Colorado, N New Mexico, Montana and North Dakota. * *villosus* (Linnaeus, 1766)—from E North Dakota E to S Quebec and Nova Scotia, and S to E Colorado, C Texas, Missouri (Ozark Plateau) and N Virginia. * *terraenovae* (Batchelder, 1908)—Newfoundland.	Forests
33	*Calidris fuscicollis*	NE Alaska and N Canada E to S Baffin I. Winters in SE South America, from CE Brazil to Tierra del Fuego.	Shorelines
34	*Charadrius semipalmatus*	Alaska and N Canada, S to C British Columbia and S Nova Scotia. Winters on coasts of North and South America, from California to Chile, and from South Carolina to Patagonia; also Bermuda, West Indies, Galapagos.	Shorelines
35	*Tyrannus vociferans*	*vociferans* (Swainson, 1826)—W & C USA (SE Montana, E Wyoming, SW South Dakota, SW Nebraska, C & S California, extreme SE Nevada, S Utah, SW & SC Colorado, N & E Arizona, New Mexico, W Texas), Mexico (Baja California, and S from W Sonora and Chihuahua to Oaxaca and Chiapas) and Guatemala. *xenopterus* (Griscom, 1934)—SW Mexico (highlands of Guerrero).	Open Woodlands
36	*Spatula discors*	North America, from S Alaska to Newfoundland, and S to C USA.	Marshes
37	*Selasphorus sasin*	*sasin* (Lesson, 1829)—coastal W USA (extreme S Oregon to California, S to Santa Barbara County); winters in SC Mexico. *sedentarius* (Grinnell, 1929)—islands off coast of S California (USA).	Open Woodlands
38	*Limosa fedoa*	*beringiae* (Gibson & Kessel, 1989)—Alaska Peninsula; winters on Pacific coast of USA from S Washington to C California. *fedoa* (Linnaeus, 1758)—C Alberta and SW Manitoba to SC Montana and W Minnesota, also SW James Bay; winters from California and the Carolinas S to Panama, occasionally occurring S to N Chile, Colombia and Venezuela.	Grasslands
39	*Dendrocygna bicolor*	S USA southwards to N and E South America, as far as N Argentina; Africa S of Sahara from Senegal to Ethiopia S to South Africa and Madagascar; Indian Subcontinent E to Burma.	Marshes
40	*Tringa incana*	Extreme NE Siberia and S Alaska E to Yukon R and NW British Columbia. Winters in SW USA and W Mexico, Ecuador and Galapagos Is; also Hawaiian Is, C & S Pacific Is to E New Guinea and NE Australia.	Shorelines
41	*Calidris himantopus*	N Alaska E to S Victoria I; W & S Hudson Bay. Winters from N Chile, Bolivia and SC Brazil to N Argentina and Uruguay; small numbers locally in S USA.	Marshes
42	*Ixoreus naevius*	*meruloides* (Swainson, 1832)—Alaska and NW Canada; non-breeding also SW USA. naevius (J. F. Gmelin, 1789)—SE Alaska S to NW California. *carlottae* (A. R. Phillips, 1991)—Queen Charlotte Is, in British Columbia (SW Canada). *godfreii* (A. R. Phillips, 1991)—interior British Columbia S to NW USA (Idaho, Montana).	Forests
43	*Rallus limicola*	* *limicola* (Vieillot, 1819)—S Canada and W, C & NE USA; winters from SW Canada and E Great Lakes through S USA and Mexico to Guatemala. * *friedmanni* (Dickermann, 1966)—volcanic belt of CS Mexico to extreme S Mexico to Guatemala. * *aequatorialis* (Sharpe, 1894)—extreme SW Colombia, and Andes of Ecuador.	Marshes
44	*Melozone crissalis*	*bullatum* (Grinnell & Swarth, 1926)—SW Oregon S to extreme NC California, in W USA. *petulans* (Grinnell & Swarth, 1926)—N California in coastal zone from Humboldt County S to Marin County. *crissale* (Vigors, 1839)—C coastal California from N Monterey County E to W edge of San Joaquin Valley, S to W Kern County and Ventura County.	Scrub
45	*Egretta rufescens*	*rufescens* (Gmelin, 1789)—S USA and E Mexico through West Indies to N Colombia and N Venezuela. *dickeyi* (van Rossem, 1926)—W Mexico.	Marshes
46	*Arenaria melanocephala*	W & S Alaska. Winters from SE Alaska to NW Mexico.	Shorelines
47	*Calidris minutilla*	Alaska through NW & NC Canada to Quebec, Newfoundland and Nova Scotia. Winters from S USA through Central America and West Indies to N Chile and CE Brazil.	Marshes
48	*Sturnella neglecta*	Breeds in Canada (from S British Columbia and NC Alberta E to SW Ontario) and S in W USA (E to Michigan, W Iowa and W Texas) to Mexico (S to N Baja California, Sonora and C plateau in Guanajuato); in winter extends farther S into Mexico and E in USA to R Mississippi and the Gulf Coast.	Grasslands
49	*Plegadis chihi*	C California and NW USA S down both coasts of Mexico, wintering S to N Central America. Also SC South America, from SE Bolivia, Paraguay and S Brazil to NC Chile, NC Argentina and Uruguay.	Marshes
50	*Tringa semipalmata*	*inornatus* (Brewster, 1887)—C Alberta and SW Manitoba S to N Colorado and W Nebraska; winters from coastal S USA to N South America, mainly on Pacific coast, S to N Chile. *semipalmatus* (Gmelin, 1789)—S New Brunswick, Atlantic and Gulf coasts of USA, and West Indies; winters from Atlantic and Gulf coasts of USA and Mexico through Central America and West Indies S to S Brazil.	Shorelines
51	*Bucephala clangula*	*clangula* (Linnaeus, 1758)—N and C Europe, Asia E through USSR and N Mongolia to Kamchatka. *americana* (Bonaparte, 1838)—Alaska to Labrador.	Lakes and Ponds
52	*Calidris alpina*	*arctica* (Schiøler, 1922)—NE Greenland; winters mainly in NW Africa. *schinzii* (C. L. Brehm, 1822)—SE Greenland and Iceland through Faeroes and British Is to Baltic and S Scandinavia; winters in S Europe and NW Africa. *alpina* (Linnaeus, 1758)—N Scandinavia and N Russia E to R Kolyma; winters from W Europe and Mediterranean to NW India, and sparsely to Bangladesh.	Shorelines
53	*Pelecanus occidentalis*	occidentalis Linnaeus, 1766—W Indies. *carolinensis* (Gmelin, 1789)—Atlantic coasts of tropical America, from S Carolina to Orinoco. *californicus* (Ridgway, 1884)—Pacific coast of America, from California to Mexico.	Oceans
54	*Chordeiles minor*	* *minor* (J. R. Forster, 1771)—most of C & S Canada S to N & NE USA. * *hesperis* (Grinnell, 1905)—SW Canada and W USA. * *sennetti* (Coues, 1888)—CS Canada and NC USA.	Grasslands
55	*Sphyrapicus nuchalis*	Rocky Mountains from SE British Columbia and SW & SE Alberta E to W South Dakota, and S, discontinuously, to SE California, E Arizona and NE & S New Mexico; in winter from S California, Arizona and W Texas S to SC Mexico.s	Forests
56	*Calidris melanotos*	Taymyr E through Chukotskiy Peninsula to W & N Alaska, NC Canada and W Hudson Bay; recent records from Yamal Peninsula, NW Siberia. Winters from S Bolivia, Paraguay and N Argentina to S Argentina; much smaller population in SE Australia, Tasmania and New Zealand.	Grasslands
57	*Phalaropus tricolor*	N Alberta and EC California E to Great Lakes area, with scattered small outlying populations. Winters from N Peru to Uruguay and S to Tierra del Fuego.	Lakes and Ponds
58	*Parkesia noveboracensis*	*notabilis* Ridgway, 1881—breeds from Alaska and NW Canada S to NW USA (EC Oregon to extreme NW Wyoming), and E to N Ontario and Great Lakes. *limnaeus* (McCabe & A. H. Miller, 1933)—breeds C British Columbia (W Canada). *noveboracensis* (J. F. Gmelin, 1789)—breeds SE Canada (E Ontario E to Newfoundland) and NE USA (S to N West Virginia).	Forests
59	*Icterus spurius*	Breeds in S Canada (S Saskatchewan, S Manitoba and extreme SE Ontario) and most of E USA (from Minnesota and Maine), S in Mexico to Jalisco, Michoacán and Hidalgo; winters from S Mexico (Colima and Veracruz) S throughout Central America to Colombia and extreme NW Venezuela.	Open Woodlands
60	*Piranga rubra*	*cooperi* (Ridgway, 1869)—breeds SW USA (from SE California, S Nevada and C Arizona E to W Texas) and N Mexico (S to to NE Baja California, Sonora, N Durango, SW Coahuila and C Nuevo León); winters C & S Mexico (from S Sinaloa S to Michoacán, Morelos and Guerrero). *rubra* (Linnaeus, 1758)—breeds E USA (from SE Nebraska, S Iowa, Ohio and Delaware S to C Texas, Gulf Coast and Florida); winters from S Mexico (Michoacán, Pueblo, Veracruz and Yucatán) S to N South America (S occasionally to N Bolivia, W Brazil and SE Venezuela, occasionally the Guianas.	Open Woodlands
61	*Pipilo maculatus*	*oregonus* (Bell, 1849)—extreme SW Canada (SW British Columbia) S in W USA to SW Oregon; non-breeding S to S California. *falcinellus* (Swarth, 1913)—interior & SW Oregon S to California (W & SE slopes of Sierra Nevada). *falcifer* (McGregor, 1900)—coastal N California (S to Santa Cruz and San Benito Counties).	Scrub
62	*Charadrius melodus*	N Great Plains, Great Lakes and Atlantic coast from Newfoundland S to North Carolina. Winters on Atlantic coast of S USA, Gulf of Mexico, Bahamas and rather locally in Greater Antilles; also W Mexico.	Shorelines
63	*Calidris pusilla*	W & N Alaska and N Canada. Winters on Pacific coast from S Mexico to S Peru, and on Atlantic coast from Yucatán and West Indies to C Argentina.	Shorelines
64	*Archilochus alexandri*	SW Canada (British Columbia) S through W USA (E to E Texas) to N Mexico (N Baja California, NE Sonora to NW Tamaulipas). Winters in W & SC Mexico (S Sonora to N Guerrero and W Veracruz).	Open Woodlands
65	*Catharus guttatus*	* *guttatus* (Pallas, 1811)—S Alaska and W Canada; non-breeding W USA and N & C Mexico. * *nanus* (Audubon, 1839)—SE Alaska and W Canada; non-breeding W USA, NW Mexico (Baja California). * *slevini* (Grinnell, 1901)—W coast of USA; non-breeding NW Mexico.	Open Woodlands
66	*Cardinalis cardinalis*	*superbus* (Ridgway, 1885)—SW USA (extreme SE California E through Arizona to SW New Mexico) S to NW Mexico (N Sonora). *townsendi* (van Rossem, 1932)—Tiburón I and adjacent coastal Sonora, in W Mexico. *affinis* (Nelson, 1899)—WC Mexico (SE Sonora, SW Chihuahua, Sinaloa and W Durango).	Open Woodlands
67	*Calidris mauri*	E Chukotskiy Peninsula and W & N Alaska. Winters on Pacific coast from N California to N Peru, and locally down Atlantic coast from S New Jersey through Gulf of Mexico and West Indies to Surinam.	Shorelines
68	*Zonotrichia leucophrys*	* *gambelii* (Nuttall, 1840)—breeds Alaska and W Canada (E to Nunavut and N Manitoba, S to S British Columbia, SW Alberta and N Saskatchewan; non-breeding quarters in S British Columbia and W USA (E to NE Kansas, S to California) and Mexico (S to Nayarit, Aguascalientes and San Luis Potosí). * *leucophrys* (J. R. Forster, 1772)—breeds in C & E Canada from N Manitoba and N Ontario E through N & NC Quebec to N Labrador and N Newfoundland; winters in S & SE USA (S from Kansas, Missouri, Kentucky and North Carolina) and N Mexico (S to Sonora). * *pugetensis* (Grinnell, 1928)—breeds extreme SW Canada (SW British Columbia) S along US coast to NW California; non-breeding S to SW California.	Scrub
69	*Leucophaeus atricilla*	*megalopterus* (Bruch, 1855)—SE California to W Mexico (Jalisco), and Nova Scotia to Florida and Texas, locally through E Central America; winters S to S Peru. *atricilla* (Linnaeus, 1758)—West Indies to Trinidad and islands off Venezuela; winters S to N Brazil.	Shorelines
70	*Larus californicus*	*albertaensis* (Jehl, 1987)—S Mackenzie (Great Slake L), Alberta and W Manitoba to South Dakota. *californicus* (Lawrence, 1854)—E Washington and Great Basin to C Montana and Wyoming.	Lakes and Ponds
71	*Chondestes grammacus*	*strigatus* (Swainson, 1827)—breeds in S Canada from S interior British Columbia and SE Alberta E to S Saskatchewan and S Manitoba, and W USA from Oregon E to the Dakotas and S to S California, C Nevada and S Arizona (Chiricahua Mts), S to N Mexico (N Sonora E to Coahuila, S to Zacatecas, Nuevo León and W Tamaulipas); non-breeding from S edge of breeding range S to S Louisiana and Mexico (S, except Yucatán Peninsula and adjacent lowlands, to S Baja California, Veracruz and Chiapas). *grammacus* (Say, 1822)—breeds from Minnesota and C Wisconsin S to Texas, E to Ohio, Mississippi Valley and Louisiana; non-breeding from C Texas and S Louisiana S through C Mexico to Guerrero and Oaxaca.	Grasslands
72	*Calidris bairdii*	Wrangel I and Chukotskiy Peninsula E across N Alaska and N Canada to Ellesmere I, N Baffin I and NW Greenland. Winters in W & S South America.	Shorelines
73	*Aechmophorus occidentalis*	*occidentalis* (Lawrence, 1858)—SW & SC Canada, W & NC USA; winters Pacific coast S to Baja California. *ephemeralis* (Dickerman, 1986)—CW & SC Mexico.	Lakes and Ponds
74	*Selasphorus rufus*	Coastal SE Alaska S through SW Canada (British Columbia, W Alberta) to NW USA (Washington, N Idaho and extreme W Montana to N California). Winters throughout most of Mexico (except NC) and along Gulf coast of USA.	Open Woodlands
75	*Athene cunicularia*	*hypugaea* (Bonaparte, 1825)—from S Canada, W of E edge of Great Plains, S to El Salvador. *rostrata* (C. H. Townsend, 1890)—Clarión I in Revillagigedo Is (off W Mexico). *floridana* (Ridgway, 1874)—Florida (also, marginally, Georgia) and Bahamas; also (possibly this race) Cuba and I of Pines.	Grasslands
76	*Leuconotopicus albolarvatus*	* *albolarvatus* (Cassin, 1850)—SC British Columbia S, discontinuously in mountains, to Washington, W Idaho, Oregon, California (S in Coast Ranges to NW Colusa, and along Sierra Nevada in E), and extreme W Nevada. * *gravirostris* (Grinnell, 1902)—SW California (mountains around Los Angeles and San Diego).	Forests
77	*Salpinctes obsoletus*	* *obsoletus* (Say, 1823)—breeds W North America from S British Columbia, S Alberta and S Saskatchewan S to coastal California (including islands of San Nicolas and San Clemente), Oklahoma and W Texas, and W, N & C Mexico from Baja California (including San Martín I) S at higher elevations to Oaxaca; N populations migrate to S parts of range. * *guadeloupensis* (Ridgway, 1876)—Guadalupe I, off W Baja California. * *tenuirostris* (van Rossem, 1943)—San Benito, off W Baja California.	Deserts
78	*Selasphorus calliope*	SW Canada (C & E British Columbia and SW Alberta) and W USA in Rocky Mts and Sierra Nevada (Washington to California and E to Wyoming and W Colorado). Winters in WC & SC Mexico (SE Sinaloa to EC Oaxaca).	Open Woodlands
79	*Empidonax difficilis*	*difficilis* (S. F. Baird, 1858)—breeds from SE Alaska S to S California (generally W of Sierra Nevadas) and extreme NW Mexico (N Baja California); winters W Mexico (S to S Baja California, Sinaloa and Oaxaca). *insulicola* (Oberholser, 1897)—breeds on Channel Is, off S California (USA); winter range unknown. *cineritius* (Brewster, 1888)—Sierra de la Laguna (Victoria Mts), in Cape District (S Baja California).	Forests
80	*Haematopus bachmani*	W Aleutian Is (Kiska I) through S Alaska (including Round I), W Canada and W USA to NW Mexico (Los Coronados Is and Baja California). Outside breeding season, mostly concentrated between S British Columbia and Baja California.	Shorelines
